# Radiofrequency ablation versus repeat hepatectomy in the treatment of recurrent hepatocellular carcinoma in subcapsular location: a retrospective cohort study

**DOI:** 10.1186/s12957-021-02277-4

**Published:** 2021-06-14

**Authors:** Fuqun Wei, Qizhen Huang, Yang Zhou, Liuping Luo, Yongyi Zeng

**Affiliations:** 1 Department of Hepatopancreatobiliary Surgery, Mengchao Hepatobiliary Hospital of Fujian Medical University, Xihong Road 312, Fuzhou, 350025 Fujian China; 2 Department of Radiation Oncology, Mengchao Hepatobiliary Hospital of Fujian Medical University, Fuzhou, China

**Keywords:** Recurrent hepatocellular carcinoma, Radiofrequency ablation, Repeat hepatectomy, Propensity-score matching, Subcapsular location

## Abstract

**Background:**

Repeat hepatectomy and radiofrequency ablation (RFA) are widely used to treat early recurrent hepatocellular carcinoma (RHCC) located in the subcapsular region, but the optimal treatment strategy remains to be controversial.

**Methods:**

A total of 126 RHCC patients in the subcapsular location after initial radical hepatectomy were included in this study between Dec 2014 and Jan 2018. These patients were divided into the RFA group (46 cases) and the repeat hepatectomy group (80 cases). The primary endpoints include repeat recurrence-free survival (rRFS) and overall survival (OS), and the secondary endpoint was complications. The propensity-score matching (PSM) was conducted to minimize the bias. Complications were evaluated using the Clavien-Dindo classification, and severe complications were defined as classification of complications of ≥grade 3.

**Results:**

There were no significant differences in the incidence of severe complications were observed between RFA group and repeat hepatectomy group in rRFS and OS both before (1-, 2-, and 3-year rRFS rates were 65.2%, 47.5%, and 33.3% vs 72.5%, 51.2%, and 39.2%, respectively, *P* = 0.48; 1-, 2-, and 3-year OS rates were 93.5%, 80.2%, and 67.9% vs 93.7%, 75.8%, and 64.2%, respectively, *P* = 0.92) and after PSM (1-, 2-, and 3-year rRFS rates were 68.6%, 51.0%, and 34.0% vs 71.4%, 42.9%, and 32.3%, respectively, *P* = 0.78; 1-, 2-, and 3-year OS rates were 94.3%, 82.9%, and 71.4% vs 88.6%, 73.8%, and 59.0%, respectively, *P* = 0.36). Moreover, no significant differences in the incidence of severe complications were observed between the RFA group and repeat hepatectomy group.

**Conclusion:**

Both repeat hepatectomy and RFA are shown to be effective and safe for the treatment of RHCC located in the subcapsular region.

**Supplementary Information:**

The online version contains supplementary material available at 10.1186/s12957-021-02277-4.

## Introduction

Hepatocellular carcinoma (HCC) is the fourth most common cause of tumor-associated death globally [1, 2]. Radical resection is the major promising curative treatments for patients with HCC. However, the recurrence rate still remained as high as 70% within 5 years after undergoing radical surgery [3]. The treatment options for recurrent HCC (RHCC) include hepatectomy, liver transplantation, local ablation, and so on. Due to the lack of donors in some countries, liver transplantation cannot be performed frequently [4]. Repeat hepatectomy and radiofrequency ablation (RFA) are more widely used for treating RHCC. A randomized clinical trial reported no significant differences in the survival rate of patients with early-stage RHCC between repeat hepatectomy and RFA [5]. However, both repeat hepatectomy and RFA had shortcomings. Some studies showed that the complications and conversion rates were increased after receiving repeat surgery in RHCC patients due to increased postoperative adhesions and risk of bowel injury [6, 7]. Although RFA has been reported to have better tolerability, less loss of blood, shorter hospital stay, and fewer perioperative complications than repeat hepatectomy [8, 9], tumor location was thought to be an influential factor affecting RFA, and this is because of close tumor location to a large vessel or liver capsule, which is potentially a high-risk location for RFA [10]. There are two major issues associated with RFA of subcapsular liver cancer: firstly, due to narrow space for electrode placement, which in turn results in not enough ablative margin along the hepatic capsule, and those patients had high local tumor progression (LTP) rate [11, 12]. Secondly, during subcapsular tumor treatment, the associated thermal injury of adjacent structures, bleeding, or tumor seeding along the tract or within the peritoneum led to an increased risk of major complications [13]. In recent years, some studies have shown that RFA can be used for subcapsular HCC [10, 14]. And there was a study that proved RFA could be as effective as liver resection [15]. But whether repeat hepatectomy or RFA is an optimal treatment for patients with subcapsular RHCC remains unknown when preoperative disease status is eligible for both repeat hepatectomy and RFA. Hence, in this study, the prognosis of subcapsular RHCC patients undergoing RFA or repeat hepatectomy was compared.

## Material and methods

### Patient selection

This study was conducted according to the ethical guidelines of the 1975 Declaration of Helsinki and was approved by the Mengchao Hepatobiliary Hospital of Fujian Medical University’s Ethics Committee (No. 2019_068_01).

**Fig. 1:**
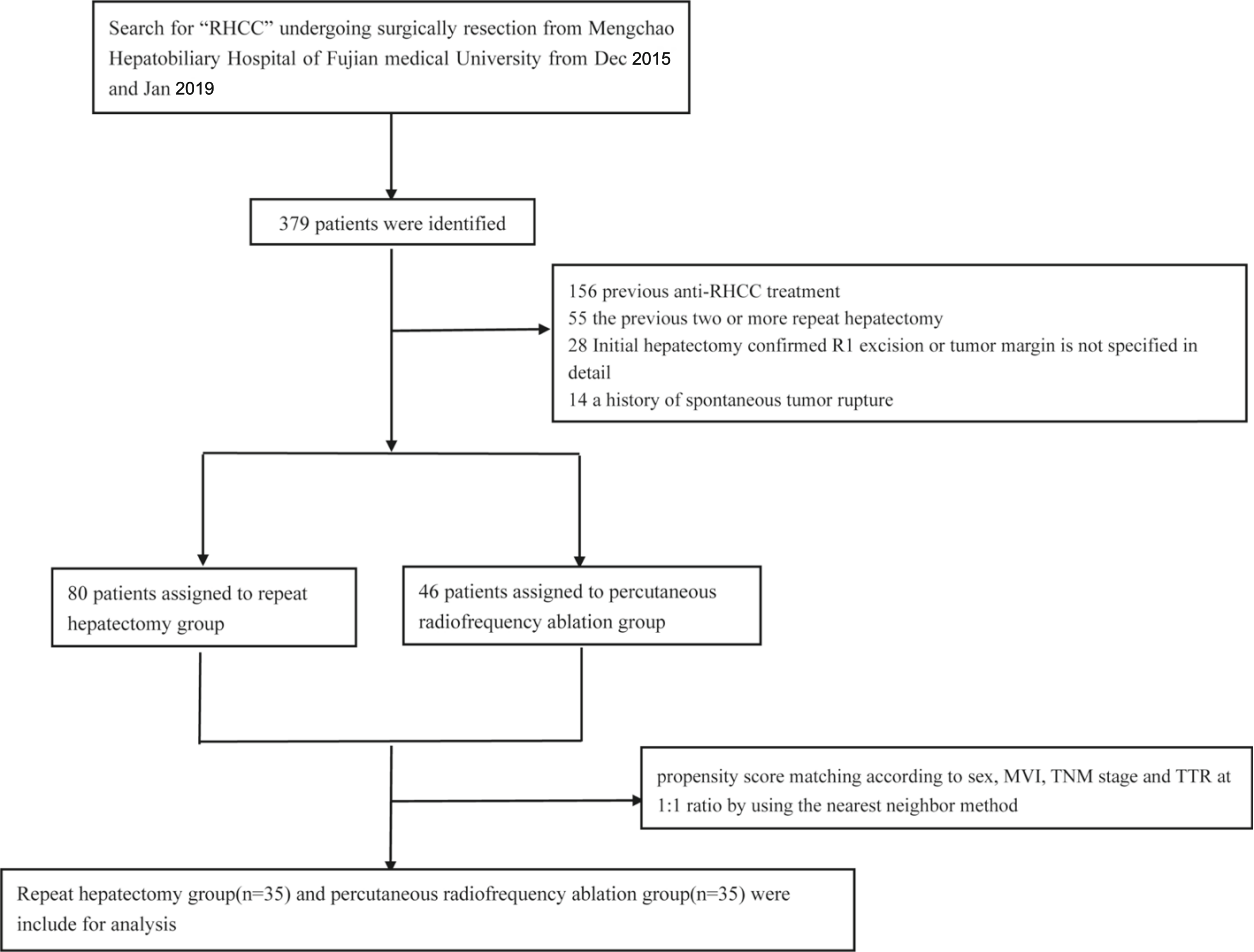
The flow chart of enrollment

Between Dec 2014 and Jan 2018, the medical records of consecutive patients with RHCC at Mengchao Hepatobiliary Hospital of Fujian Medical University were reviewed. The inclusion criteria were as follows: patients (1) who underwent radical resection as initial treatment and diagnosed with HCC by pathology, (2) who were diagnosed with RHCC based on the criteria of diagnosis of HCC of the European Association for the Study of the Liver [16], (3) with a solitary RHCC nodule (≤5cm in diameter) or less than 3 nodules (each ≤ 3 cm), (4) with first recurrence of RHCC after radical operation, (5) with no macroscopic vascular invasion and extrahepatic distant metastasis, (6) with a performance status of 0~1, and (7) of Child-Pugh class A. RHCC is defined as the presence of tumor in the subcapsular region by two radiologists after reaching a consensus [17, 18], and subcapsular HCC was defined as an index tumor that abutted the liver capsule on axial or coronal computed tomography (CT) and/or magnetic resonance images (distance from the hepatic capsule to the tumor margin is 0.1 cm). The exclusion criteria were as follows: patients (1) who received two or more repeat hepatectomy previously, (2) who had second or more recurrences after radical operation, (3) who were confirmed with R1 excision or that tumor margin is unclear by postoperative pathology, (4) with a history of other malignant neoplasms within the past 5 years, (5) with a history of spontaneous tumor rupture, (6) who received any previous anti-RHCC treatments, (7) who had recurrent tumors not under the capsule, (8) who lost to follow-up or had no further imaging studies performed after the intervention, and (9) with severe concomitant diseases, acute or active infectious diseases, and pregnancy or breastfeeding women. The flow chart of the enrollment process is presented in Fig. 1.

### Data collection

The medical data of initial resection and secondary treatment of RHCC patients were retrospectively collected from our hospital, and this includes demographics, RHCC-related factors, preoperative serum biochemistry data, preoperative serum alpha fetoprotein (AFP) level, preoperative serum protein induced by vitamin K absence or antagonist-II (des-gamma-carboxy-prothrombin (DCP)) level, imaging characteristics of tumors judged by preoperative imaging findings (including maximum tumor size, tumor number, tumor location, and presence of vascular invasion), pathological results of initial HCC [including different grades of tumors, hepatic capsule, microvascular invasion (MVI)], surgery-related factors including the extent of liver resection (major liver resection or minor liver resection), intraoperative bleeding, and intraoperative blood transfusion.

### RFA

RFA was chosen when patients with subcapsular RHCC recurrence were not willing to undergo a second operation or considered that RFA could achieve the same effect after multidisciplinary discussion. RFA was performed by radiologists with more than 5 years of experience in interventional therapy during the start of this study. The RFA procedure was conducted according to the RITA system (RITA StarBurst FLEX, RITA Medical Systems, Mountain View, CA, USA). The patient’s cardiovascular and respiratory systems were continuously monitored when conducting the procedure. RFA was conducted under the guidance of ultrasonography or CT and achieved an adequate safety margin of 0.5~1.0 cm if possible. Sometimes, to ensure that the radiofrequency electrode needle covers the capsule side of the tumor for complete ablation, repeat ablation is considered necessary.

### Repeat hepatectomy

Hepatectomy was performed by surgeons with at least 10 years of experience in liver surgery. The type of surgery was decided according to a routine discussion for each patient in the department of liver surgery. The procedure done was as follows: (1) the abdominal cavity, liver, and adjacent organs were thoroughly explored. If necessary, the tumor can be probed with intraoperative ultrasound. Surgical resection area and resection method were determined by comprehensive evaluation of tumor size, tumor location, and local blood supply; (2) the perihepatic anadesma was cut off, the liver was dissociated to fully expose the tumor, and the hepatic vein should be carefully treated when dissociating precisely a part of the right liver; (3) when performing anatomic hepatectomy, the corresponding liver pedicle was first dissected and then blocked. In case of irregular hepatectomy, the main vessels of the tumor from the donor should be dissected first, clipped, and then cut off. Anatomical resection of the left liver and massive liver resection were performed by dissecting the second hilum as far as possible to pre-block the corresponding hepatic vein to reduce reflux bleeding. A conventional preset hilar occlusion band was used to control accidental intraoperative bleeding. In some special patients who need to be blocked for a long time, the block can be divided into 10min each with 5-min interval; (4) the liver was cut off with an ultrasonic scalpel (HAR36, Ethicon EndoSurgery, USA), and the aspirator assisted in attracting and exposing the operative field. In case of larger blood vessels or bile ducts, then the structure of the pipeline should be dissected first and cut off with linear cutter reload (75mm, Ethicon EndoSurgery, USA) clips or Hemolock linear cutter reload (LT300, Ethicon EndoSurgery, USA) clips. Endo-GIA (45mm, Covidien, USA) was used in the treatment of hepatic pedicle or main hepatic vein. Bipolar electrocoagulation or electrocoagulation hook was used to treat punctate bleeding and bleeding of liver cross-section; (5) the section of the liver was covered with hemostatic gauze after no bleeding or bile leakage. An abdominal drainage tube was indwelled conventionally, and the tumor was removed out to confirm whether the tumor was completely removed. Radical resection was performed in the patient who not only underwent R0 resection but also had no recurrence within 2 months after resection according to the Chinese guidelines [19].

### Follow-up and recurrence treatment

All patients who received repeat hepatectomy or RFA were prospectively followed up using serum tumor marker levels as well as ultrasonography, contrast-enhanced computed tomography (CE-CT), or magnetic resonance imaging (MRI) of the abdomen at intervals of 2 to 3 months during the first year after the operation and 3 to 6 months later. Chest CT examination and bone scintigraphy were performed when extrahepatic RHCC was suspected. Positron emission tomography (PET) CT might be used to detect the occult metastatic disease. Overall survival (OS) was defined as the interval from repeat treatment to death due to any cause, and it was censored at the date of the last follow-up visit when the patients were still alive. Time to recurrence <12 months was defined as early recurrence [20]. Repeat recurrence-free survival (rRFS) was defined as the interval between repeated treatment and the first documented HCC recurrence or death. Repeat recurrence was managed according to a patient’s general performance, liver function, degree of cirrhosis, size, number of nodules, and location of the repeat recurrent tumor [21, 22].

### Propensity-score matching (PSM)

PSM R version 3.6 (http://www.r-project.org/) was applied to achieve balanced exposure groups at baseline, and potentially confounding factors included MVI, AFP level, recurrent tumor number, and time to recurrence. The patients were matched by a 1:1 ratio using the nearest neighbor method with a caliper of 0.2.

### Statistics

Comparison of categorical data was carried out with Pearson χ^2^ test, and the forward method of univariate and multivariate Cox regression analysis was used for evaluating rRFS and OS before and after PSM to determine independent prognostic factors and clinically recognized prognostic factors were included in multivariate Cox regression analysis. The OS and rRFS were calculated with the Kaplan-Meier method. *P* < 0.05 was considered statistically significant, and all data were analyzed with SPSS 22.0.

## Results

### Clinical characteristics of all patients

A total of 379 RHCC patients underwent repeat treatment or RFA between 2014 and 2018, but only 126 patients were included according to the inclusion criteria and all patients were hepatitis B virus positive. The details of patient selection were summarized in the flow chart (Fig. 1). Before PSM, higher MVI incidence, higher tumor differentiation grade, larger recurrent tumor, more multiple tumors, and more early recurrence were observed in the RFA group (all *P* < 0.05). After PSM, no significant differences in the baseline characteristics were observed between the two groups (all *P* > 0.05, Supplementary Table 1).

### Repeat recurrence-free survival and overall survival

**Fig. 2:**
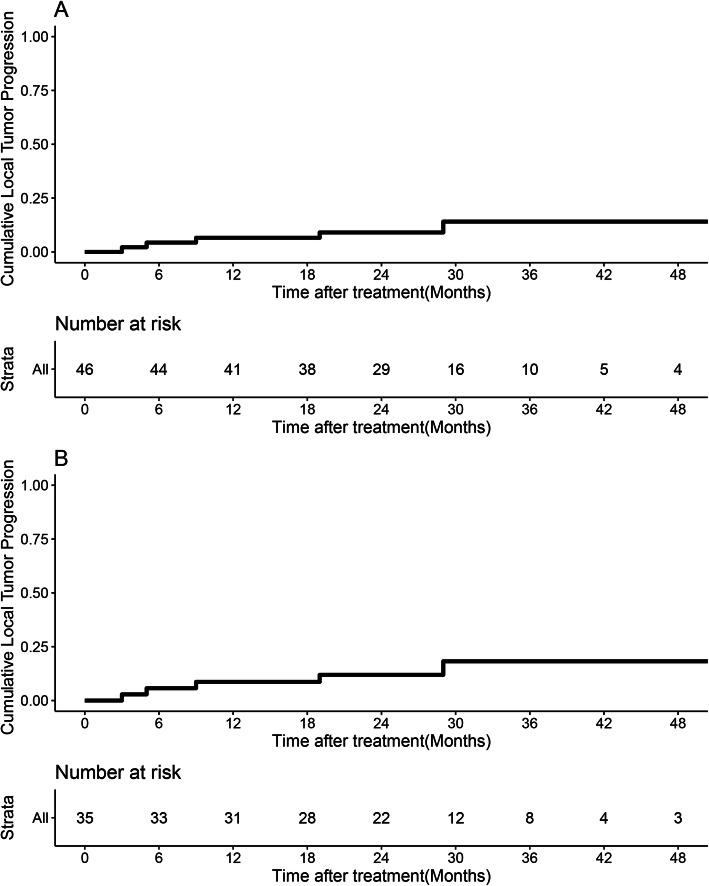
Cumulative LTP rate and Kaplan-Meier OS rate curves generated before and after propensity-score matching in the RFA group. **A** Before PSM and **B** after PSM

**Fig. 3:**
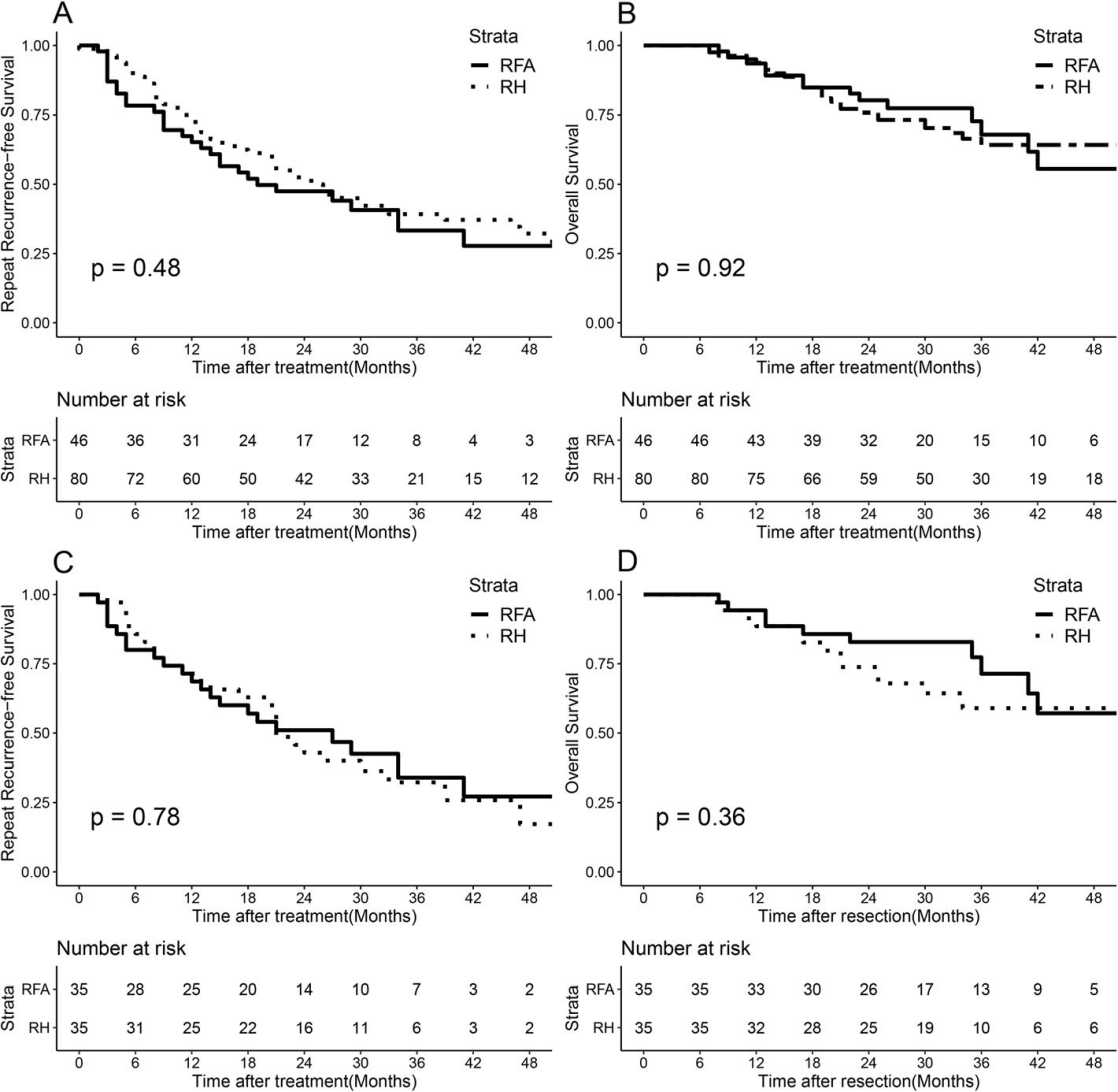
Survival curves of all patients with recurrent hepatocellular carcinoma who underwent radiofrequency ablation and repeat hepatectomy groups. **A** Cumulative repeat disease-free survival (rRFS) curves and **B** cumulative overall survival (OS) curves before propensity-score matching. **C** Cumulative rRFS curves and **D** cumulative OS curves after propensity-score matching

The 6-month, 1-, 2-, and 3-year LTP rates in the RFA group were 2.5%, 6.6%, 9%, and 14.1%, respectively, (Fig. 2A) and were changed to 5.7%, 8.7%, 11.9%, and 18.2% after PSM analysis (Fig. 2B). The median follow-up time was 31.0 (range, 7.0–63.0) months. Before PSM, the median rRFS of the RFA group was 18.0 (range, 4.0–54.0) months, which was similar to that of the repeat hepatectomy group [25.4 (range, 3.0–61.0) months]. The 1-, 2-, and 3-year rRFS rates were 65.2%, 47.5%, and 33.3% in the RFA group, respectively, and 72.5%, 51.2%, and 39.2% in the repeat hepatectomy group, respectively (*P* = 0.48, Fig. 3A). The median OS of the RFA group was 27.25 (range, 8.0–63.0) months, which was similar to that of the repeat hepatectomy group [32.22 (range, 7.0–61.0) months]. The 1-year, 2-year, and 3-year OS rates were 93.5%, 80.2%, and 67.9% for the RFA group, and 93.7%, 75.8%, and 64.2% for the repeat hepatectomy group, respectively (*P* = 0.92, Fig. 3B).

After PSM, the median rRFS of the RFA group was 20.33 (range, 3.0–53.0) months, which was similar to that of the repeat hepatectomy group [22.20 (range, 3.0–58.0) months]. The 1-, 2-, and 3-year rRFS rates were 68.6%, 51.0%, and 34.0% for the RFA group, and 71.4%, 42.9%, and 32.3% for the repeat hepatectomy group, respectively (*P* = 0.78, Fig. 3C). The median OS in the RFA group was 29.0 (range, 8.0–63.0) months, which was similar to that in the repeat hepatectomy group [30.0 (range, 7.0–58.0) months]. The 1-, 2-, and 3-year OS rates were 94.3%, 82.9%, and 71.4% in the RFA group, and 88.6%, 73.8%, and 59.0% in the repeat hepatectomy group, respectively (*P* = 0.36, Fig. 3D).

### Univariate and multivariate analyses of rRFS and OS for patients before and after PSM

The results of univariate and multivariate Cox regression analyses of rRFS and OS for RHCC after curative resection before PSM are presented in Table 1. Before PSM, univariate analysis showed that the MVI and multiple recurrent tumors showed an association with increased tumor recurrence rate (*P* < 0.05). Multivariate Cox regression analysis revealed MVI and larger recurrent tumor size as independent risk factors of rRFS (*P* < 0.05). For OS, the univariate analysis showed that narrow tumor margin, MVI, the advanced TNM stage, highly recurrent AFP levels, and early recurrence showed association with shorter OS (all *P* < 0.05), and multivariate Cox regression analysis identified MVI as the only independent risk factor of OS.

**Table 1:** Univariate and multivariable analyses of rRFS and OS in all patients before PSM

Variable	rRFS						OS					
	Univariate			Multivariate			Univariate			Multivariate		
	HR	95% CI	*P*	HR	95% CI	*P*	HR	95% CI	*P*	HR	95% CI	*P*
Initial hepatectomy stage data												
Age (≤45y vs >45y)	0.809	0.492–1.330	0.403	1.207	0.566–2.574	0.626	0.919	0.452–1.867	0.816	2.783	0.618–12.539	0.183
Gender (male vs female)	1.185	0.611–2.299	0.616	0.630	0.360–1.097	0.103	4.004	0.963–16.646	0.056	0.841	0.372–1.900	0.677
HBV-DNA (<500 vs ≥500 IU/ML)	1.179	0.753–1.848	0.472				1.601	0.878–2.919	0.124			
AFP level (<200 vs ≥200ng/ml)	1.418	0.893–2.252	0.139	0.791	0.441–1.417	0.430	1.812	0.982–3.345	0.054	0.785	0.334–1.845	0.578
DCP median (mAU/ML)	2.063	1.336–3.181	0.841				1.075	1.706–2.461	0.055			
Blood loss (ml)	1.001	1.000–1.002	0.095				1.000	0.999–1.002	0.841			
Indocyanine green (%)	0.988	0.957–1.020	0.467				0.959	0.910–1.011	0.118			
Maximum tumor size (<3 vs ≥3cm)	1.350	0.855–2.130	0.198				1.722	0.897–3.307	0.103			
Tumor number (single vs multiple)	1.296	0.803–2.091	0.289				1.687	0.899–3.165	0.104			
Tumor capsule (complete vs incomplete)	1.116	0.715–1.743	0.628				1.030	0.557–1.093	0.925			
Tumor margin (<1 vs ≥1cm)	0.721	0.463–1.124	0.149	0.812	0.487–1.353	0.424	0.497	0.269–0.919	0.026*	0.487	0.236–1.004	0.051
Differentiation grade (I/II vs III/IV)	0.889	0.507–1.558	0.680				1.084	0.520–2.261	0.829			
MVI (no vs yes)	1.840	1.170–2.893	0.008*	1.820	1.082–3.060	0.024*	2.289	1.244–4.212	0.008*	2.025	1.021–4.016	0.043*
8th TNM stage (IA + IB vs II + IIIA)	0.897	0.606–1.276	0.499	0.665	0.403–1.099	0.111	1.621	1.047–2.510	0.030*	1.690	0.914–3.123	0.094
Extent of liver resection (major vs minor)	0.751	0.439–1.284	0.295				0.507	0.214–1.204	0.124			
Recurrent stage data												
AFP level (<200 vs ≥200ng/ml)	1.153	0.904–2.532	0.115	1.413	0.704–2.837	0.331	2.531	1.334–4.801	0.004*	2.204	0.787–5.206	0.143
Maximum recurrent tumor size (<3 vs 3~5cm)	1.692	0.815–3.513	0.158	2.804	1.180–6.663	0.020*	1.696	0.666–4.317	0.268	2.238	0.765–6.546	0.141
Recurrent tumor number (single vs multiple)	1.881	1.115–3.171	0.018*	2.791	1.249–6.236	0.102	1.811	0.968–3.390	0.063	2.118	0.807–5.554	0.127
TTR (≤12 vs >12m)	0.715	0.461–1.110	0.135	0.861	0.518–1.431	0.564	0.479	0.261–0.879	0.017*	0.573	0.278–1.180	0.131
Treatment strategy (repeat hepatectomy vs RFA)	0.818	0.520–1.287	0.385	0.925	0.498–1.718	0.806	0.779	0.497–1.223	0.279	1.010	0.525–1.944	0.976

*Abbreviations*: *AFP*, α-fetoprotein; *CI*, confidence interval; *DCP*, des-γ-carboxy-prothrombin; *HR*, hazard ratio; *MVI*, microvascular invasion; *TTR*, time to recurrence; *RFA*, radiofrequency ablation. *P < 0.05

After PSM, univariate analysis showed that high DCP level, narrow tumor margin, and larger recurrent tumor size were associated with increased tumor recurrence rate (*P* < 0.05) but multivariate Cox regression analysis showed none of them as an independent risk factor. For OS, univariate analysis showed that narrow tumor margin, high recurrent AFP level, larger recurrent tumor size, and early recurrence showed association with shorter OS (all *P* < 0.05), while initial AFP level, tumor margin, MVI, and TTR were shown to be as independent risk factors of OS (Table 2).

**Table 2:** Univariate and multivariate analyses of rRFS and OS in all patients after PSM

Variable	rRFS						OS					
	Univariate			Multivariate			Univariate			Multivariate		
	HR	95% CI	*P*	HR	95% CI	*P*	HR	95% CI	*P*	HR	95% CI	*P*
Initial hepatectomy stage data												
Age (≤45y vs >45y)	0.914	0.464–1.799	0.794	1.463	0.440–4.870	0.535	1.484	0.508–4.335	0.470	5.437	0.434–8.173	0.189
Gender (male vs female)	1.398	0.549–3.556	0.482	0.669	0.232–1.928	0.457	4.647	0.622–34.730	0.134	0.622	0.113–3.417	0.585
HBV-DNA (<500 vs ≥500 IU/ML)	1.134	0.624–2.061	0.679				1.223	0.549–2.725	0.622			
AFP level (<200 vs ≥200ng/ml)	1.570	0.844–2.920	0.154	0.629	0.245–1.614	0.335	1.266	0.545–2.941	0.583	0.282	0.081–0.986	0.048*
DCP median (mAU/ML)	1.001	0.768–1.181	0.047*	1.003	0.998–1.008	0.244	1.001	0.706–1461	0.458	1.094	0.546–4.098	0.654
Blood loss (ml)	1.001	0.999–1.002	0.336				1.000	0.998–1.002	0.829			
Indocyanine green (%)	0.981	0.936–1.028	0.427				0.958	0.887–1.036	0.284			
Maximum tumor size (<3 vs ≥3cm)	1.113	0.620–2.072	0.684				1.735	0.723–4.165	0.218			
Tumor number (single vs multiple)	1.617	0.861–3.039	0.135				1.857	0.796–4.331	0.152			
Tumor capsule (complete vs incomplete)	1.157	0.644–2.078	0.626				0.608	0.252–1.465	0.267			
Tumor margin (<1 vs ≥1cm)	0.059	0.283–0.914	0.024*	0.491	0.209–1.152	0.102	0.304	0.135–0.688	0.004*	0.161	0.048–0.533	0.003*
Differentiation grade (I/II vs III/IV)	0.810	0.411–1.597	0.542				1.161	0.484–2.784	0.739			
MVI (no vs yes)	1.454	0.809–2.614	0.211	1.616	0.827–3.157	0.160	2.029	0.917–4.491	0.081	4.002	1.201–13.332	0.024*
8th TNM stage (IA + IB vs II + IIIA)	0.794	0.491–1.286	0.349	0.598	0.272–1.135	0.201	1.629	0.940–2.824	0.082	1.904	0.743–4.877	0.180
Extent of liver resection (major vs minor)	0.683	0.317–1.474	0.332				0.529	0.157–1.777	0.304			
Recurrent stage data												
AFP level (<200 vs ≥200ng/ml)	1.934	0.988–3.786	0.054	1.128	0.416–3.060	0.813	3.198	1.369–7.467	0.007*	0.385	0.095–1.566	0.182
Maximum recurrent tumor size (<3 vs 3~5cm)	5.806	2.224–15.158	0.000*	3.237	0.682–15.396	0.139	4.282	1.429–12.837	0.009*	2.454	0.424–14.213	0.317
Recurrent tumor number (single vs multiple)	1.743	0.873–3.479	0.115	3.137	0.754–13.051	0.116	1.284	0.444–3.714	0.644	6.702	0.677–18.080	0.104
TTR (≤12 vs >12m)	0.742	0.413–1.335	0.320	0.759	0.316–1.862	0.539	0.392	0.157–0.982	0.046*	0.198	0.048–0.826	0.026*
Treatment strategy (repeat hepatectomy vs RFA)	1.084	0.610–1.925	0.784	0.958	0.429–2.136	0.916	1.440	0.652–3.180	0.367	1.536	0.595–3.965	0.375

*Abbreviations*: *AFP*, α-fetoprotein; *DCP*, des-γ-carboxy-prothrombin; *MVI*, microvascular invasion; *TTR*, time to recurrence; *RFA*, radiofrequency ablation. *P < 0.05

### Complications

There was no difference in the incidence of Clavien-Dindo classification grade 3 or 4 complications between the RFA group and repeat hepatectomy group (*P* = 0.627). Ascites, pleural effusion, wound or puncture site infection, subdiaphragmatic fluid collection, bile leakage, intra-abdominal hemorrhage, pneumonia, upper gastrointestinal tract bleeding, and atelectasis were similar between the two groups (all *P* > 0.05), while high fever morbidity (*P* = 0.011) was observed in the repeat hepatectomy group, and more hepatic subcapsular hematoma (*P* = 0.007) and pneumothorax (*P* = 0.021) were observed in the RFA group (Table 3).

**Table 3:** Complications after repeat hepatectomy and RFA

Complications	Repeat hepatectomy, no. (%) (n = 80)	RFA, no. (%) (n = 46)	*P* value
Fever (>38.5°C, >3 d)	14 (17.5)	1 (2.2)	0.011*
Ascites	3 (3.8)	0	0.184
Pleural effusion	5 (6.3)	3 (6.5)	0.952
Postoperative liver failure	0	0	–
Wound or puncture site infection	5 (6.3)	0	0.084
Subdiaphragmatic fluid collection	3 (3.8)	1 (2.2)	0.672
Bile leakage	2 (2.5)	0	0.280
Intra-abdominal hemorrhage	1 (1.3)	1 (2.2)	0.690
Pneumonia	5 (6.3)	1 (2.2)	0.301
Upper gastrointestinal tract bleeding	0	1 (2.2)	0.185
Atelectasis	4 (5)	0	0.123
Atrial fibrillation	0	0	–
Ileus	0	0	–
Hepatic subcapsular hematoma	0	4 (8.7)	0.007*
Pneumothorax	0	3(6.5)	0.021*
Grade 3 or 4 complication	3 (3.8)	1 (2.2)	0.627

*P < 0.05

## Discussion

The incidence of high recurrence is the primary cause of poor prognosis of HCC [16]. Repeat hepatectomy for RHCC has been considered as the best curative treatment till date because of the limitations and shortcomings associated with the alternative radiofrequency ablation (RFA), transcatheter arterial chemo-embolization (TACE), and systemic chemotherapy [23–27]. Nevertheless, the surgical procedure of repeat hepatectomy remains challenging and may increase complications of postoperative ascites and decompensation of liver function. The current study indicated that RFA seems to be as effective as repeat hepatectomy for the treatment of RHCC and also has merits of being less invasive, highly target selective, and repeatable [28–30]. Generally, when conducting the RFA procedure, the tumor location, particularly in the poor efficiency area such as the upper part of the gallbladder, gastrointestinal tract, and diaphragm, should be taken into consideration [31–33]. Due to these HCC locations, RFA of subcapsular tumors has two significant problems that incomplete ablation and high incidence of complications [34]. For the above two methods, there is still debate as to which approach is better. This study concluded no statistically significant differences in the OS and rRFS among patients with RHCC located at the subcapsular who underwent repeat hepatectomy or RFA before and after PSM. The incidence of severe complications after repeat hepatectomy was similar to those after RFA. Notably, the repeat hepatectomy group was more likely to have febrile complications due to anesthetically induced atelectasis and surgically induced release of inflammatory factors than the RFA group. However, subcapsular hematoma and pneumothorax were more common in the RFA group. The selection of specific treatment methods for RHCC still needs multidisciplinary discussion. Patients with subcapsular RHCC of less than 3cm and early relapse refused to undergo reoperation or accompanied with severe liver cirrhosis, and it is recommended to choose RFA to reduce complications and hospital stay to achieve a similar therapeutic effect by surgery. Other patients suggest to choose repeat surgery to reduce the incidence of LTP because subcapsular RHCC still has a 10 to 15% incidence of LTP under RFA.

Previous studies [35–40] have illustrated tumor size and tumor number as independent risk factors of postoperative survival, and RHCC is no exception. In this study, multiple recurrent tumors and large recurrent tumor size affect the prognosis of RHCC, and the selection of management methods for such subcapsular RHCC requires further clinical studies. A western strategy [41] emphasizes the impact of pathologic profile of the first resection in RHCC, satellitosis, and MVI at initial resection as negative prognostic factors of survival after recurrence in this research, while our study also showed that patients with MVI at the time of initial resection had poor OS. A multicenter PSM analysis [42] indicated that repeat hepatectomy for early relapse showed association with worse OS and disease-free survival when compared with late relapse. In our study, patients who relapsed late had better survival rates than those who relapsed early. The reason may be that patients with early recurrence are likely to have recessive micrometastases.

However, there are some limitations in our study. Firstly, this was a retrospective study based on a single center, and therefore, selection bias can be hardly avoided, despite PSM was carried out. Secondly, several confounding factors related to efficacy cannot be compared in the two groups, such as the differences of recurrent tumor capsule, recurrent tumor differentiation, and MVI, which remained to be unknown. Thirdly, patients with other chronic liver diseases such as hepatitis C and alcoholic liver disease were not included in this study. Fourthly, this paper lacks a large enough sample size and the follow-up period was not long enough.

## Conclusion

In summary, the prognosis and the incidence of severe complications associated with RHCC in the subcapsular location after RFA or repeat hepatectomy showed no significant difference. Further studies, such as random multicenter research, for a more effective multidisciplinary treatment strategy for RHCC are warranted.

## Supplementary Information

**Additional file 1: Supplementary Table 1.** Baseline characteristics before and after propensity score matching.

## Data Availability

The datasets generated and/or analyzed during the current study are not publicly available due to hospital policy but are available from the corresponding author on reasonable request.
