# Supplementary material for: Radiofrequency ablation versus repeat hepatectomy in the treatment of recurrent hepatocellular carcinoma in subcapsular location: a retrospective cohort study
Source: World J Surg Oncol. 2021 Jun 14;19:175. doi: 10.1186/s12957-021-02277-4 (PMC8204439; doi:10.1186/s12957-021-02277-4)
Supplement: Supplementary file 1 — Additional file 1: Supplementary Table 1. Baseline characteristics before and after propensity score matching. [file 12957_2021_2277_MOESM1_ESM.docx]

**Supplementary Table 1. Baseline characteristics before and after propensity score matching**

| Variable | Before matching | | |  | After matching | | |
| --- | --- | --- | --- | --- | --- | --- | --- |
|  | RFA | Repeat hepatectomy | *P* value |  | RFA | Repeat hepatectomy | *P* value |
| Initial hepatectomy stage data |  |  |  |  |  |  |  |
| Age |  |  |  |  |  |  |  |
| ≤45y | 9 (19.6%) | 20 (25.0%) | 0.633 |  | 5 (14.3%) | 10 (28.6%) | 0.244 |
| >45y | 37 (80.4%) | 60 (75.0%) |  |  | 30 (85.7%) | 25 (71.4%) |  |
| Gender |  |  |  |  |  |  |  |
| Female | 6 (13.0%) | 10 (12.5%) | 1.000 |  | 4 (11.4%) | 4 (11.4%) | 1.000 |
| Male | 40 (87.0%) | 70 (87.5%) |  |  | 31 (88.6%) | 31 (88.6%) |  |
| HBV-DNA , IU/ML |  |  |  |  |  |  |  |
| <500 | 30 (65.2%) | 49 (61.2%) | 0.801 |  | 23 (65.7%) | 22 (62.9%) | 1.000 |
| ≥500 | 16 (34.8%) | 31 (38.8%) |  |  | 12 (34.3%) | 13 (37.1%) |  |
| Baseline laboratory investigations | |  |  |  |  |  |  |
| ALT, mean±SD, IU/L | 39.77 ±25.25 | 35.37 ±22.65 | 0.315 |  | 38.0±22.26 | 38.81±27.88 | 0.894 |
| AST, mean±SD,IU/L | 41.47 ±28.50 | 34.44 ±20.62 | 0.112 |  | 37.78±20.12 | 34.07±16.73 | 0.406 |
| GGT, mean±SD,IU/L | 71.87 ±78.73 | 53.43 ±41.83 | 0.088 |  | 78.49±88.67 | 59.06±50.88 | 0.265 |
| Total bilirubin,mean±SD, μmol/L | 15.28 ±6.88 | 18.04 ±8.32 | 0.058 |  | 15.20±7.33 | 18.271±0.51 | 0.161 |
| Albumin, mean±SD, g/L | 39.28 | 39.09 ±4.01 | 0.799 |  | 39.60±3.87 | 38.60±4.26 | 0.307 |
| Platelet,mean±SD, ×109/L | 146.62±61.65 | 157.19 ±58.80 | 0.340 |  | 144.40±59.66 | 160.14±50.74 | 0.239 |
| Prothrombin time, mean±SD, s | 13.68±0.95 | 16.05 ±19.50 | 0.406 |  | 13.64±1.01 | 13.79±0.93 | 0.526 |
| AFP level, ng/mL |  |  |  |  |  |  |  |
| <200 | 31 (67.4%) | 59 (73.8%) | 0.578 |  | 23 (65.7%) | 28 (80.0%) | 0.282 |
| ≥200 | 15 (32.6%) | 21 (26.2%) |  |  | 12 (34.3%) | 7 (20.0%) |  |
| DCP, mean±SD, mAU/ML | 4673.66±15559.38 | 2742.89±10973.62 | 0.584 |  | 1358.36±3848.15 | 2948.23±10566.04 | 0.418 |
| Blood loss, mean±SD , ml | 220.43±259.29 | 164.18 ±156.71 | 0.163 |  | 212.86±264.40 | 184.86±169.07 | 0.600 |
| Indocyanine green  mean ±SD, % | 8.04±7.52 | 8.09 ±6.88 | 0.971 |  | 8.45±7.68 | 7.00±4.97 | 0.355 |
| Maximum tumor size, cm |  |  |  |  |  |  |  |
| <3 | 17 (37.0%) | 33 (41.2%) | 0.776 |  | 13 (37.1%) | 13 (37.1%) | 1.000 |
| ≥3 | 29 (63.0%) | 47 (58.8%) |  |  | 22 (62.9%) | 22 (62.9%) |  |
| Tumor number, n (%) |  |  |  |  |  |  |  |
| Single | 32 (69.6%) | 61 (76.2%) | 0.541 |  | 23 (65.7%) | 29 (82.9%) | 0.172 |
| Multiple | 14 (30.4%) | 19 (23.8%) |  |  | 12 (34.3%) | 6 (17.1%) |  |
| Tumor capsule, n (%) |  |  |  |  |  |  |  |
| Incomplete | 28 (60.9%) | 51 (63.8%) | 0.896 |  | 23 (65.7%) | 22 (62.9%) | 1.000 |
| Complete | 18 (39.1%) | 29 (36.2%) |  |  | 12 (34.3%) | 13 (37.1%) |  |
| Tumor margin, cm,n (%) |  |  |  |  |  |  |  |
| <1 | 17(36.9%) | 34(42.5%) | 0.765 |  | 13(37.1%) | 14(40.0%) | 0.807 |
| ≥1 | 29(63.1%) | 46(57.5%) |  |  | 22(62.9%) | 21(60.0%) |  |
| Tumor differentiation grade, n (%) |  |  |  |  |  |  |  |
| I/II | 32 (69.6%) | 68 (85.0%) | 0.066 |  | 29 (82.9%) | 24 (68.6%) | 0.265 |
| III/IV | 14 (30.4%) | 12 (15.0%) |  |  | 6 (17.1%) | 11 (31.4%) |  |
| MVI, n (%) |  |  |  |  |  |  |  |
| No | 25 (54.3%) | 59 (73.8%) | 0.042* |  | 18 (51.4%) | 22 (62.9%) | 0.469 |
| Yes | 21 (45.7%) | 21 (26.2%) |  |  | 17 (48.6%) | 13 (37.1%) |  |
| Extent of liver resection, n (%) |  |  |  |  |  |  |  |
| Major | 9 (19.6%) | 18 (22.5%) | 0.872 |  | 6 (17.1%) | 6 (17.1%) | 1.000 |
| Minor | 37 (80.4%) | 62 (77.5%) |  |  | 29 (82.9%) | 29 (82.9%) |  |
| 8th TNM stage, n (%) |  |  |  |  |  |  |  |
| IA+IB | 36 (78.3%) | 52 (65.0%) | 0.295 |  | 25 (71.4%) | 22 (62.9%) | 0.747 |
| II | 7 (15.2%) | 20 (25.0%) |  |  | 7 (20.0%) | 9 (25.7%) |  |
| IIIA | 3 (6.5%) | 8 (10.0%) |  |  | 3 (8.6%) | 4 (11.4%) |  |
| Hospital stay, days | 5.8±1.48 | 10±3.08 | 0.025* |  | 5.6±1.34 | 9.8±3.45 | 0.034* |
| Costs, Chinese Yuan | 20408.8±1057.2 | 37245.2±570.0 | 0.000* |  | 20008.8±537.7 | 37045.2±493.0 | 0.000* |
| **Recurrent stage data** |  |  |  |  |  |  |  |
| AFP level, ng/mL |  |  |  |  |  |  |  |
| <200 | 42 (91.3%) | 60 (75.0%) | 0.029* |  | 31(88.6%) | 25(71.4%) | 0.073 |
| ≥200 | 4 (8.7%) | 20 (25.0%) |  |  | 4(11.4%) | 10(28.6%) |  |
| Maximum recurrent tumor size, cm |  |  |  |  |  |  |  |
| <3 | 43(93.5%) | 71(88.8%) | 0.472 |  | 32(91.4%) | 33(94.3%) | 0.643 |
| 3~5 | 3(6.5%) | 9(11.2%) |  |  | 3(8.6%) | 2(5.7%) |  |
| Recurrent tumor number |  |  |  |  |  |  |  |
| Single | 33(71.7%) | 69(86.3%) | 0.018* |  | 24(68.6%) | 30(85.7%) | 0.088 |
| Multiple | 13(28.3%) | 11(13.7%) |  |  | 11(31.4%) | 5(14.3%) |  |
| TTR, n (%) |  |  |  |  |  |  |  |
| ≤12m | 28 (60.9%) | 25 (31.2%) | 0.002* |  | 21 (60.0%) | 19 (54.3%) | 0.809 |
| >12m | 18 (39.1%) | 55 (68.8%) |  |  | 14 (40.0%) | 16 (45.7%) |  |

Abbreviations: ALT, alanine aminotransferase; AST, aspartate transaminase; GGT,γ-Glutamyl Transferase; AFP, α‐fetoprotein; DCP, des-γ-carboxy-prothrombin; MVI, microvascular invasion; TTR, time to recurrence.* *P*<0.05.
